# Generation of renal tubular organoids from adult SOX9^+^ kidney progenitor cells

**DOI:** 10.1093/lifemedi/lnad047

**Published:** 2023-11-23

**Authors:** Dewei Zhou, Dandan Li, Hao Nie, Jun Duan, Sarah Liu, Yujia Wang, Wei Zuo

**Affiliations:** Laboratory of Transplant Engineering and Transplant Immunology, West China Hospital, Sichuan University, Chengdu 610041, China; Department of Regenerative Medicine, Shanghai East Hospital, Tongji University School of Medicine, Shanghai 200120, China; Department of Regenerative Medicine, Shanghai East Hospital, Tongji University School of Medicine, Shanghai 200120, China; Department of Regenerative Medicine, Shanghai East Hospital, Tongji University School of Medicine, Shanghai 200120, China; Department of Regenerative Medicine, Shanghai East Hospital, Tongji University School of Medicine, Shanghai 200120, China; Super Organ R&D Center, Regend Therapeutics, Shanghai 201210, China; Department of Regenerative Medicine, Shanghai East Hospital, Tongji University School of Medicine, Shanghai 200120, China; Super Organ R&D Center, Regend Therapeutics, Shanghai 201210, China; Laboratory of Transplant Engineering and Transplant Immunology, West China Hospital, Sichuan University, Chengdu 610041, China; Department of Regenerative Medicine, Shanghai East Hospital, Tongji University School of Medicine, Shanghai 200120, China; Super Organ R&D Center, Regend Therapeutics, Shanghai 201210, China

**Keywords:** kidney organoids, single-cell sequencing, kidney progenitor cells, renal tubular, acute kidney injury

## Abstract

The pathogenesis of several kidney diseases results in the eventual destruction of the renal tubular system, which can progress to end-stage renal disease. Previous studies have demonstrated the involvement of a population of SOX9-positive cells in kidney regeneration and repair process following kidney injury. However, the ability of these cells to autonomously generate kidney organoids has never been investigated. Here, we isolated SOX9^+^ kidney progenitor cells (KPCs) from both mice and humans and tested their differentiation potential *in vitro*. The data showed that the human SOX9^+^ KPC could self-assemble into organoids with kidney-like morphology. We also used single-cell RNA sequencing to characterize the organoid cell populations and identified four distinct types of renal tubular cells. Compared to the induced pluripotent stem cell-derived kidney organoids, KPC demonstrated more tubular differentiation potential but failed to differentiate into glomerular cells. KPC-derived organoid formation involved the expression of genes related to metanephric development and followed a similar mechanism to renal injury repair in acute kidney injury patients. Altogether, our study provided a potentially useful approach to generating kidney tubular organoids for future application.

## Introduction

In 2009, Hans Clever’s group pioneered using a single mouse LGR5^+^ intestinal stem cell to self-organize into an intestinal organoid, exhibiting an intestinal crypt-villus structure *in vitro* [1]. Since then, organoids have obtained increasing attention as invaluable tools for studying human diseases, conducting *in vitro* drug screening, and exploring tissue replacement in various organs including kidney [2]. Human kidneys are made up of a million nephrons as filtering units, and each nephron includes a glomerulus and a tubule. To generate kidney organoids, in 2015, a comprehensive protocol was established for the direct differentiation of human pluripotent stem cells into complex multicellular kidney organoids, serving as a foundation for subsequent refinements and improvements by numerous researchers [2–7]. Embryonic stem cell (ESC) and induced pluripotent stem cell (iPSC)-derived kidney organoids have been instrumental in recapitulating the pathogenesis of kidney diseases, including glomerular disease and autosomal dominant polycystic kidney disease [8–12]. Moreover, these organoids are suitable for drug screening due to their size and cellular complexity, enabling more effective screening procedures [13]. However, ESC/iPSC-derived kidney organoids are characterized by their structural complexity, which leads to a very low proportion of tubule cells and hinders the study of renal tubular tissue [12]. Furthermore, the limited ability to fully replicate embryonic developmental processes *in vitro* resulted in the generation of non-renal cells, such as neural and muscle cells, that are irrelevant to the target organ [14, 15]. To address this issue, other groups proposed using adult renal cells to generate organoids with reduced complexity compared to pluripotent stem cell (PSC)-derived organoids [16].

Adult SOX9^+^ renal cells, which have the potential to play a dominant role in tissue repair following acute kidney injury, have been a subject of debate regarding their origin—whether they arise from dedifferentiation of surviving epithelial cells or represent a distinct resident epithelial cell population [17–20]. Recently, our group has successfully isolated SOX9^+^ kidney progenitor cells (KPCs) from healthy human urine and demonstrated their ability to differentiate into proximal epithelial cells resembling renal tubules *in vivo* [21]. In the current study, we demonstrated the capacity of SOX9^+^ KPCs to undergo stable and directed differentiation into renal tubular cells, resulting in the self-organization of renal tubular organoids *in vitro*. Analysis of single-cell sequencing (scRNA-seq) data revealed the presence of three types of immature tubular tissue in the organoids: proximal tubule, loop of Henle, and collecting duct. The data revealed that renal tubular organoids exhibit functionality in maintaining ion concentration stability and regulating body fluid levels. Therefore, our study identified a novel cell type capable of renal tubular organoids formation.

## Result

### Clone SOX9^+^ progenitor cells from adult mouse kidney

**Figure 1.**
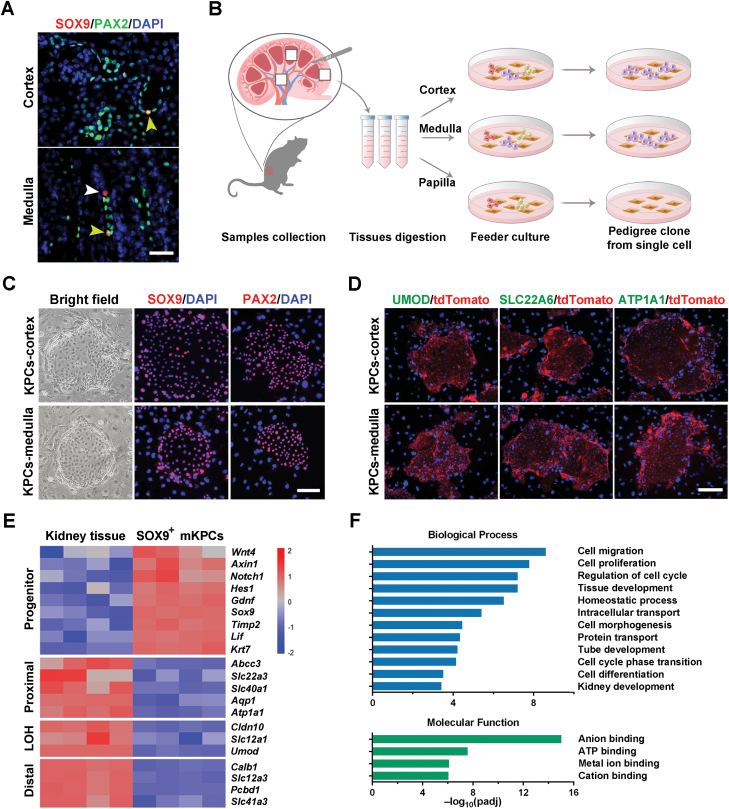
**Clone and transcriptomic analysis of mouse kidney progenitor cells.** (A) Representative images of mouse kidney immunofluorescence staining for SOX9 and PAX2. The arrowheads indicate SOX9^+^PAX2^+^ cells or SOX9^+^ PAX2^−^ cells. (Scale bar: 50 μm) (B) Schematic illustrates the process of selective culture of kidney progenitor cells derived from the cortex, medulla, and papilla of mouse kidneys. (C) Representative images of mouse KPCs immunofluorescence staining for SOX9 and PAX2. (Scale bar: 50 μm) (D) Representative images of mouse KPCs immunofluorescence staining for UMOD, SLC22A6, and ATP1A1. Cells were derived from tdTomato-expressing mice. (Scale bar: 50 μm) (E) Heatmap of differentially expressed gene set exhibiting a distinct transcriptome between mouse kidney tissue and the corresponding SOX9^+^ clonogenic KPCs by microarray analysis. Duplicates were taken from independent biological samples. (F) GO enrichment analysis of differentially expressed genes in medulla-derived renal progenitors versus mouse medulla tissue.

First, we investigated the localization of SOX9^+^ cells in the adult mouse kidney. Immunofluorescence staining of healthy mice kidneys revealed that SOX9^+^ cells were predominantly located in the renal tubular region (Fig. 1A). To culture and isolate progenitor cells from the kidneys of healthy mice, we dissected tissue from the cortex, medulla, and papilla of normal adult mice kidneys and digested them into single-cell suspensions for regenerative cell cloning (R-Clone) culture system (Fig. 1B). Following 3–7 days of culturing on feeder cells, we successfully obtained compact epithelial clones from the cortex and medulla tissue, but not from the papilla tissue. The specificity of the distribution areas confirmed that SOX9^+^ cells primarily act in repairing renal tubules [20].

We conducted a detailed immunofluorescent analysis on the cell clones, comparing SOX9-specific antibodies with various antibody identifiers of key cell types in the mouse kidney. Immunostaining verified the expression of both *Sox9* and the essential transcription factor for kidney development, *Pax2*, in these cells [22–24] (Figs. 1C, S1A and S1C). Fluorescence intensity analysis showed that KPCs in different groups had stable average fluorescence intensity (Fig. S1B and S1D). In contrast, the cell clone did not show the expression of mature renal tubular markers (Figs. 1D and S1E). Therefore, we speculated that the cloned SOX9^+^ cells as mouse-derived KPCs. We also performed transcriptomic profile analysis of mouse KPCs and renal tissue cells. The analysis revealed no significant expression differences between cortex-derived and medulla-derived KPCs, suggesting that the cell distributed in the two regions were similar. However, compared to mature renal tissue, KPCs from both regions showed high expression of genes that are crucial in prenatal renal development, including *Sox9*, *Lif*, *Gdnf*, and *Krt7* [25, 26]. The KPCs also exhibited a signature for core signalling pathway genes, such as Wnt (*Wnt4*, *Axin1*) and Notch (*Notch1*, *Hes1*) pathway genes [27, 28] (Fig. 1E). In contrast, the KPCs did not express genes specific to mature renal tubules or glomeruli, including those for proximal tubules (*Aqp1*, *Atp1a1*), loop of Henle (LOH) (*Slc12a1*, *Umod*), and distal tubules (*Slc12a3*). Additionally, transcriptome and Gene Ontology (GO) enrichment analysis revealed that the function of KPCs was primarily involved in cell proliferation, tissue development, and cell differentiation (Fig. 1F).

### Clone SOX9^+^ kidney progenitor cells from adult human kidney and urine

**Figure 2.**
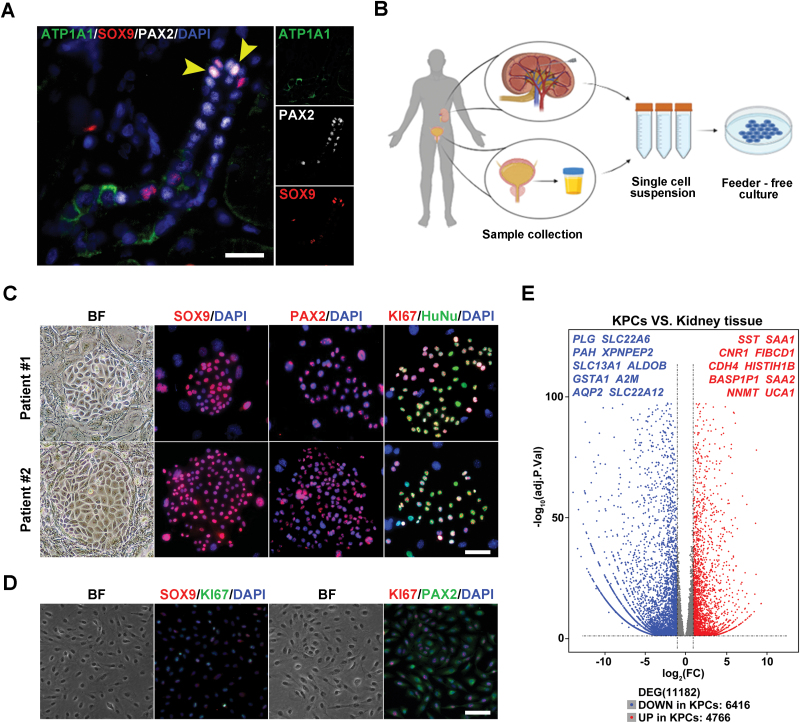
**Clone and transcriptomic analysis of human kidney progenitor cells.** (A) Representative images of adult human kidney immunofluorescence staining for SOX9, ATP1A1, and PAX2. Arrowheads indicate SOX9^+^ PAX2^+^ cells. (Scale bar: 50 μm) (B) Schematic showed the isolate process of kidney progenitor cells from human kidney and urine. (Scale bar: 50 μm) (C) Representative immunofluorescence staining images of patient KPC colonies for SOX9, PAX2, KI67, and HuNu. (Scale bar: 50 μm) (D) Representative images of human KPCs immunofluorescence staining for SOX9, PAX2, and KI67. KPCs were derived from cultures in a feeder-free condition. (Scale bar: 50 μm) (E) Volcano plot of up-regulated and down-regulated gene expression showing significant differences in human KPCs versus human kidney tissue with top-ranked genes listed.

For the human kidney, we also identified a population of SOX9^+^ PAX2^+^ co-labelled cells located in tubule niches that did not express the tubule-specific marker ATP1A1 (Fig. 2A). Here, we attempted to isolate progenitor cells from both human kidney tissue and urine samples (Fig. 2B). Single-cell suspensions obtained from needle kidney biopsy tissue of patients with chronic kidney diseases (CKD) were seeded for cell culture, yielding clones consisting of proliferative (KI67^+^) cells that expressed SOX9 and PAX2 (Fig. 2C). These cells could be maintained for large-scale expansion. Meanwhile, following the separation steps of previous experiments, we collected urine samples from a healthy donor and successfully isolated cell clones. Immunofluorescence staining showed that the cell clones expressed *SOX9*, *PAX2*, and proliferating marker *KI67* (Figs. 2D and S2A). Fluorescence intensity analysis showed that SOX9, PAX2, and KI67 were stably expressed in KPCs (Fig. S2B). To minimize the impact of feeder cells in subsequent experiments, we attempted to culture KPCs without feeder cells. Now we have successfully adapted the human KPCs from feeder-based to feeder-free conditions, where the cell lines proliferated steadily for at least eight passages.

Furthermore, using RNA-seq analysis, we identified several novel markers of progenitor cells, as illustrated in the volcano plot (Fig. 2E). Among these markers, two serum amyloid A genes (*SAA1* and *SAA2*) ranked highly in the expressed gene list. Previous reports have shown that the *SAA* gene can ‘reprogram’ rat tubule cell lines into regenerative cells [29]. In addition, we also discovered new cell surface genes, including *SST*, *CNR1*, and *CDH4*. Further research is required to validate the potential of these new markers for facilitating flow sorting and analysis of kidney progenitor cells.

### Mouse- and human-derived KPCs constructed 3D renal tubular organoids

**Figure 3.**
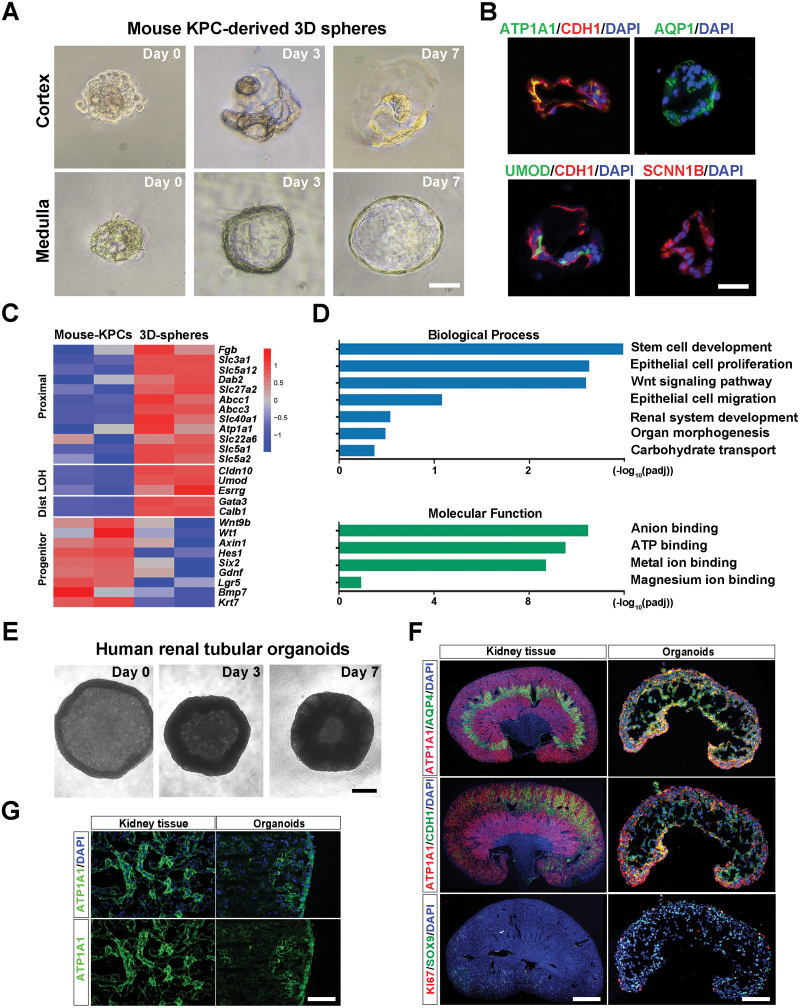
**Generation of mouse 3D spheroids and human renal tubular organoids.** (A) The process of construction of mouse-derived spheres. (Scale bar: 100 μm) (B) Representative images of mouse-derived spheres immunofluorescence staining for ATP1A1, CDH1, AQP1, UMOD, and SCNN1B. (Scale bar: 100 μm) (C) Heatmap of differentially expressed genes exhibiting a distinct transcriptome between KPCs (medulla-derived) and the KPC-assembled spheres. Duplicates were taken from independent biological samples. (D) GO enrichment analysis of genes highly expressed in medulla-derived spheres. (E) The process of formation of human-derived renal tubular organoids. (Scale bar: 100 μm) (F) Representative images of immunofluorescence staining for ATP1A1, AQP4, CDH1, SOX9, and KI67. Left: mouse kidney (Scale bar: 500 μm); right: Human-derived renal tubular organoids (Scale bar: 100 μm). (G) Representative images of immunofluorescence staining for ATP1A1. Left: mouse kidney; right: Human-derived renal tubular organoids. (Scale bar: 25 μm)

Previous studies have demonstrated that human KPCs can differentiate into mature renal tubular tissue *in vivo* [21]. However, whether KPCs can form organoids *in vitro* remains to be studied. Here, we performed *in vitro* differentiation test cultures of mouse and human KPCs. First of all, we investigated the differentiation potential of mouse SOX9^+^ KPCs in a three-dimensional (3D) sphere culture system. By removing stemness-maintenance signals and supplementing with retinoic acid (RA)/fibroblast growth factor 9 (FGF9)/hepatocyte growth factor (HGF) [30, 31], we found that mouse KPCs demonstrated robust differentiation capability. Within seven days of culturing, the cells spontaneously aggregated to form hollow spheres (Fig. 3A). Immunostaining of sphere sections revealed the potential for developing mature kidney tubular structures, as evidenced by the presence of mature tubule markers, including ATP1A1, AQP1, UMOD, and SCNN1B (Fig. 3B). Notably, no up-regulation of glomerular marker genes was observed in the spheres, suggesting that SOX9^+^ KPCs only have the committed tubular fate *in vitro*. To further characterize the cell identity of the 3D kidney spheres, we analysed their gene expression profiles. Compared to mouse KPCs, the 3D spheres demonstrated down-regulation of multiple progenitor-related genes (*Krt7*, *Lgr5*, *Six2*) and up-regulation of distinct tubule marker genes (*Atp1a1*, *Umod*) (Fig. 3C), consistent with the immunostaining results. GO analysis revealed the enrichment of related biological processes, including ‘Renal system development’, ‘Epithelial cell migration’, and ‘Organ morphogenesis’ in the kidney spheres. Enriched molecular functions of the sphere genes included ‘Anion binding’ and ‘Metal ion binding’, which are required for the ion transport function of renal tubules (Fig. 3D).

Next, we explored the ability of human KPCs to differentiate and form organoids. We seeded KPCs at 10^5^ cells/well density in 96-well U-bottom low cell-binding plates for 3D differentiation culture with differentiation medium. After 10 days of culture, the cells aggregated to form organoids with a ‘kidney-like’ shape (Fig. 3E). We probed a panel of essential markers at various tubule segments to compare the protein expression patterns in organoids and renal tissue [32] (Fig. 3F). We found that the proximal tubular marker ATP1A1 exhibited high binding at the outer edge and outlined a distinct tubular structure, similar to kidney tissue (Fig. 3G). CDH1, which marks the loop of Henle and distal tubules, was mainly expressed in the middle of the organoids and partly co-located with ATP1A1. Additionally, we detected the collecting duct relative marker AQP4, which was relatively more expressed in the inner medullary collecting duct. Quantitative analysis showed that the positive fluorescence area ratio of ATP1A1, CDH1, and AQP4 was about 11.2%, 14.4%, and 16.5% of the renal organoid tissue sections, and they had stable fluorescence intensity (Fig. S3A and S3B). Interestingly, the progenitor marker SOX9 was widely expressed in organoids, while less than 0.1% of cells with high proliferative capacity (KI67^+^) expressed it. Quantitative mRNA analysis showed that SOX9^+^ KPCs assembled organoids returned to a quiescent state with 5- to 15-fold upregulation of mature tubule markers (Fig. S3C). We also compared the differences between low and high passage number KPCs-induced organoids. There were small expression differences between organoids induced from different passage number of KPCs (Fig. S3D).

### scRNA-seq analysis of human-derived renal tubular organoids

**Figure 4.**
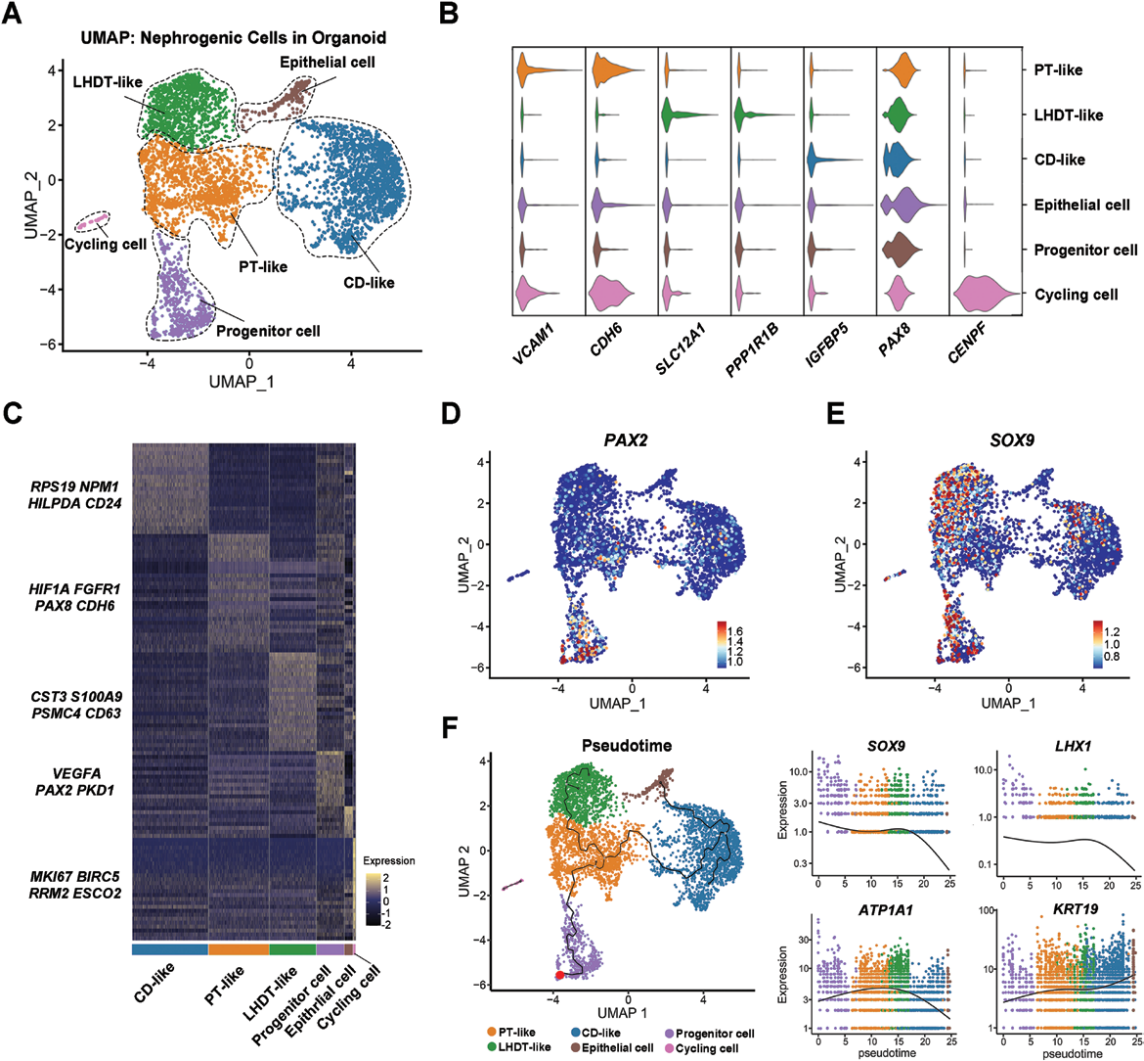
**Single-cell RNA sequencing analysis of the tubular organoids.**(A) UMAP plot showed the unsupervised of human renal tubular organoids. (B) Violin plots presented each cluster’s marker genes and highlighted the selected marker genes for each cluster. (C) Genetic heatmap showed some of the characteristic genes in each cluster. (D, E) Feature plots showed the expression of *PAX2* and *SOX9* genes in organoids. (F) Pseudotime diagram with clustering information as a reference. Feature plots showed *SOX9*, *LHX1*, *ATP1A1*, and *KRT19* genes expression varies with pseudotime series.

We performed scRNA-seq on renal tubular organoids formed by human KPCs to explore their transcriptional characteristics at the single-cell level. After stringent quality controls, we sequenced 5668 cells from the organoids. We identified six clusters from the organoids based on established cell type-specific markers and annotated their identity (Fig. 4A). Violin plots and heatmaps were used to show representative differentially expressed genes from each population (Fig. 4B and 4C), which included proximal tubular-like (PT-like) cell [33–36], loop of Henle and distal tubule-like (LHDT-like) cell [37–40], collecting duct-like (CD-like) cell [41, 42], other epithelial cell, progenitor cell, and cycling cell. The genes used to define clusters were all from the available literature (Table S1). Notably, *VCAM1*, an incomplete repair marker, was shown to be limited to the parietal epithelial layer surrounding PODXL^+^ cells. Therefore, the VCAM1^+^ cluster was identified as the proinflammatory and profibrotic proximal tubular cell in this case [43]. Additionally, progenitor cell showed a more pronounced expression of *PAX2* and *SOX9* genes, suggesting that this cluster was in the early stages of tubular cell development (Fig. 4D and 4E). Of note, although all clusters were annotated as renal tubular fate commitment, many typical mature renal tubule markers were not expressed specifically.

To explore the cellular differentiation trajectory, we performed pseudotime analysis using Monocle3 program. We assumed the progenitor cells as the starting point of differentiation (Fig. 4F). KPCs first differentiated into PT-like cells and then differentiated in different directions. One branch was LHDT-like cells and the other branch was CD-like cells. Feature plots were generated to show the genes that change with pseudotime, revealing a gradual decrease in the expression of genes representing kidney development as KPCs differentiate and mature (Fig. 4F). The expression level of the *SOX9* was highest in progenitor cell and decreased in CD-like cluster. Similarly, the progenitor cells expressed *LHX1*, and its expression also exhibited a decreasing trend, indicating gradual cell differentiation and maturation. In contrast, the genes representing mature renal tubules showed up-regulation in their respective specific tissues. While the expression of *ATP1A1* was not fully indicative of cell characterization, it remained highest in PT-like cluster. And the *KRT19* expression was highest in the CD-like and epithelial cell cluster.

### Compare iPSC-derived versus KPC-derived renal tubular organoids

**Figure 5.**
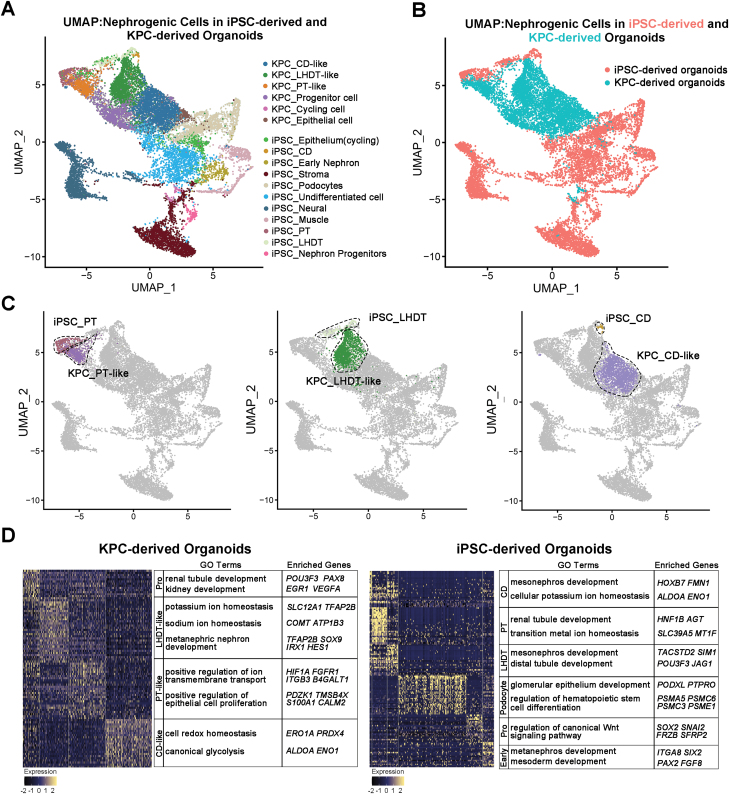
**Comparative analysis of KPC- and iPSC-derived organoids.** (A) UMAP of integrated nephrogenic cells from iPSC-derived organoids and KPC-derived organoids, colored by clusters. (B) UMAP of integrated nephrogenic cells from iPSC-derived organoids and KPC-derived organoids, coloured by their origins. (C) Compared the positional relationships of the three tubular types of cell populations in UMAP. (D) GO enrichment analysis of KPC-derived organoids and iPSC-derived organoids.

To compare the human KPC-derived renal tubular organoids cells with iPSC-derived kidney organoids, we obtained a dataset of E25 differentiation iPSC-organoids and merged the datasets [44] with ours. Cluster identification validated the presence of nephrogenic cell types (Fig. 5A) in both datasets. By comparison, we found that the differentiation direction of KPC-derived organoids was more homogeneous compared with iPSCs, which mainly differentiated towards tubules but did not differentiate into the stroma, neural or other non-renal cells (Fig. 5B). A more significant proportion of tubular cells were generated, with immature proximal tubules, distal tubules and collecting ducts accounting for 9.2%, 28.3%, and 39.6% in KPC-derived organoids, compared with 3.9%, 2.2%, and 0.4%, respectively, in iPSC-derived organoids (Fig. S4A).

We analysed the spatial position relationship of cell clusters. KPC_PT-like cells were found to be closely related to iPSC_proximal tubule cells (iPSC_PT), while KPC_LHDT-like cells exhibited proximity to iPSC_loop of Henle and distal tubule cells (iPSC_ LHDT). These results indicated the similar differentiation degrees of cells. The distance between iPSC_collecting duct cells (iPSC_CD) and KPC_CD-like was relatively far, which indicated the low differentiation degree of KPC_CD-like cells (Fig. 5C). In theory, uniform manifold approximation and projection can reflect the differentiation trajectory of cells. The KPC organoid cell population was located between the undifferentiated iPSC cells and the mature kidney cells, which indicated that the KPC organoid cells were in the state before the mature cells (Fig. 5A). These findings suggest that KPCs possess a comparable advantage for tubule differentiation and share developmental trajectory consistency with iPSCs in kidney development.

**Figure 6.**
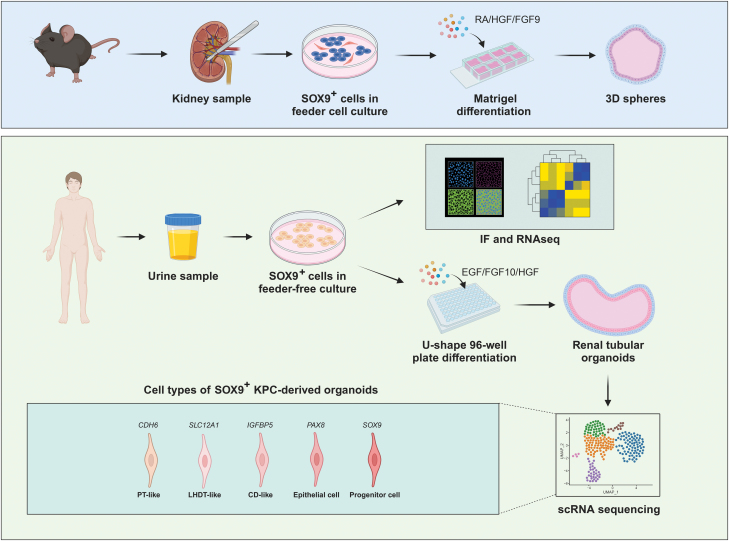
**Graphical summary of the research of adult SOX9**^**+**^ **KPC-derived renal tubular organoids.** The cartoon was created with BioRender.

By conducting GO analysis of the organoids derived from the two cell types, we observed distinct functional differences (Fig. 5D). The GO analysis revealed that the organoids derived from iPSCs exhibited enrichment in mesoderm development, mesonephros development, metanephros development, and renal tubule development, indicating a comprehensive developmental process. In contrast, the organoids derived from KPCs primarily showed associations with metanephric nephron development, renal tubule development, and kidney development, with little involvement in the germ layer developmental process. Compared with iPSC-derived organoids, KPC-derived organoids differentiated into metanephric tissue specifically and avoided contamination with pronephric or mesonephric cells [45]. Based on these findings, we hypothesized that the genes governing the *in vitro* differentiation of KPCs are primarily associated with metanephros development-related pathway genes. Moreover, we observed the enrichment of genes associated with renal function, including ‘Positive regulation of ion transmembrane transport’, ‘Potassium ion homeostasis’, and ‘Sodium ion homeostasis’ in KPC-derived organoids. By Gene Set Enrichment Analysis (GSEA) analysis, we identified the downregulation of genes related to cell proliferation and the upregulation of pathway genes associated with cellular metal ion homeostasis and regulation of body fluid levels in tubule-like cell populations (Fig. S4B). These data suggested that the KPC-derived renal tubular organoids could exhibit certain renal functions. The overall findings of the current work were summarized in Fig. 6.

## Discussion

Techniques for inducing kidney-like mini organs using iPSCs and ESCs have been well established in the past decades. Importantly, recent research has employed a scalable organoids model for studying polycystic kidney disease [13]. However, iPSC-based differentiation has certain limitations, including the need for stringent cell culture conditions and lengthy differentiation cycles. Moreover, the formation of organoid cells in iPSC cultures may lead to lack of tubular cells and the presence of impure cells, which can impact subsequent studies [15]. In light of these challenges, we developed a novel differentiation model to address these concerns and explore an alternative method for constructing kidney organoids. Specifically, we attempted to generate kidney organoids using SOX9^+^ KPCs isolated from mouse kidneys and adult human urine, and we present here the single-cell transcriptomic dataset of KPC-derived organoids. Our experimental findings provide initial evidence of the potential of kidney progenitor cells derived from kidney tissue and urine to form kidney organoids *in vitro*.

Our previous study demonstrated the ability of SOX9^+^ KPCs to differentiate into mature renal tubules *in vivo*. In this study, we have established a novel organoid culture system utilizing these cells. Both mouse and human SOX9^+^ KPCs isolated from kidney tissue or urine samples could undergo stable and long-term proliferation in a unique laboratory R-Clone culture system. When subjected to 3D differentiation in a specific medium, KPCs exhibited a tendency to differentiate and express genes associated with kidney development, including *LHX1* and *PAX2*. Morphologically, the organoids were similar to a mini kidney. Through scRNA-seq analysis, we identified three potential renal tubule-like cells in the organoid. The cells did not express typical markers of mature renal tubules but expressed some atypical marker genes of renal tubules. We speculated that the organoids were not fully mature during the culture process.

GO enrichment analysis of the differentially expressed genes among PT-like, LHDT-like, and CD-like clusters identified different segments of renal tubules enriched in various GO terms. The genes enriched in the LHDT-like group were mainly related to maintaining sodium and potassium ion homeostasis, which was consistent with the function of distal renal tubules in the kidney, indicating that renal tubular organoids may have acquired some specific biological functions. Vascular endothelial growth factor A (VEGFA) and angiogenesis-related genes were also enriched in renal tubular organoids, indicating that organoids may have the potential to recruit blood vessels, if they were transplanted *in vivo* in future studies. Many early renal and metanephric development functions were enriched in the progenitor cells cluster. This was consistent with the view that these cells were in the early stages of tubular development. A study has revealed that the metanephros arises from the posterior intermediate mesoderm, whereas the ureteric bud and the pro/mesonephros are derived from the anterior intermediate mesoderm [4]. Compared with iPSC-derived organoids, KPC-derived renal tubular organoids could differentiated into metanephric tissue.

## Research limitations

Limitations of the work include the following points: firstly, the differentiation status of renal tubular organoids according to the scRNA-seq analysis was not fully mature—only tubule-like cell was detected. One of the reasons for this may be that the differentiation time of 7 days was not enough for them to differentiate into mature renal tubule-like tissue. Secondly, the organoid culture condition based on current methods will lead the organoids into hypoxia status. We speculated that this was related to the high oxygen demand and enriched mitochondria number of tubular cells. As described in other articles, the organoids enriched many hypoxia-related genes due to the lack of a blood circulation system resulting in inadequate cellular oxygen supply to renal tubular cells [46, 47]. This requires further research and further optimization of culture conditions.

## Methods

### Culture of clonogenic mouse kidney progenitor cells

All animal studies were performed under the guidance of University Association for Laboratory Animal Care and Use. For mouse kidney progenitors cloning, kidneys of 8−10-week-old normal or mT/mG mice bred in a specific pathogen-free facility were collected to obtain renal cortex, medulla, and papilla samples. Kidney tissue acquisition was performed after the mice were euthanized. Harvested tissues were washed with ice-cold buffer (F12 medium containing 1% Pen/Strep and 5% FBS) and minced by sterile scalpel into 0.2−0.5 mm^3^ sizes to a viscous and homogeneous appearance. The minced tissue was then digested with dissociation buffer including DMEM/F12 (Gibco, USA), 2 mg/mL protease XIV (Sigma, USA), 0.01% trypsin (Gibco, USA), and 10 ng/mL DNase I (Sigma, USA) in room temperature overnight with gentle agitation. Digested cell suspensions were washed 5 times with wash buffer and passed through a 70-μm Nylon mesh (Falcon, USA) to remove aggregates. Cell pellets were collected by centrifuge of 200 g and then seeded onto a feeder layer of lethally irradiated 3T3-J2 cells in modified SCM-6F8 media as previously described. Cells were cultured at 37°C in 7.5% CO_2_ incubator. The culture media was replaced every 2 or 3 days. Colonies were digested by 0.25% trypsin-EDTA solution (Gibco, USA) for 3−5 min and passaged every 7 to 10 days. Approximately 10,000 progenitor cells were seeded to each well of a 6-well plate. For better visualization of colony growth, tdTomato^+^ KPCs derived from mT/mG mice were used.

### Isolation and culture of human kidney progenitor cells

For method of KPCs isolated from renal biopsies, patients with the indicated CKD were diagnosed by K/DOQI clinical practice guidelines and all individuals had thorough medical examinations prior to sampling. Percutaneous renal needle biopsies were performed to obtain patient kidney samples; patients were in a prone position during a biopsy. Biopsies were carried out after local anaesthesia with ultrasound-guided core tissue biopsy needles (18 gauge). All renal specimens were subjected to pathological diagnosis. All the human tissues were obtained following clinical SOP under the patient’s consent and approved by the Hospital Ethics Committee.

To isolate KPCs from urine, we collected 50−100 mL middle stream urine samples from the disease-free donor on a bland diet for at least 24 h in the morning (not the ‘first morning’). Samples were centrifuged immediately at 390 g and discarded the supernatant. The cell pellets from one subject were resuspended in ice-cold urine wash buffer (F12 medium, 5% FBS, 1% penicilin/streptomycin, 1% l-glutamine, 20 μg/mL gentamicin and 2.5 μg/mL amphotericin B) and pooled into one 50 mL conical tube, followed by extensive washing with urine wash buffer for five times and resuspended in cold phosphate-buffered saline. Cell pellets were collected and then seeded onto a feeder layer of lethally irradiated 3T3-J2 cells with modified SCM-6F8 medium in one well of a 12-well plate. Most urine-derived cells did not attach to the feeder layer and were removed by the first medium change on day 3. Single-cell colonies were selected for pedigree cloning using cloning cylinders and high vacuum grease. Cells were cultured for approximately five to six passages, and the 3T3-J2 cells were gradually removed using gradient digestion.

### Three-dimensional culture and renal tubular organoids differentiation

Matrigel differentiation assays were performed as previously described [48]. 3D culture of cells was performed on Matrigel Matrix (Corning, USA). For mouse kidney spheres formation, RA (500 nM), HGF (30 ng/mL), and FGF9 (100 ng/mL) were added to the basic differentiation medium to optimize tubular epithelium differentiation. After formation, the spheres were harvested on the 7th day, embedded in Tissue-Tek O.C.T. Compound (Sakura, USA), and frozen in a −80°C refrigerator. Five micormetre sections were obtained and subjected to immunofluorescence for the indicated mature markers.

The differentiation of human renal tubular organoids referred to the iPSC-induced construction of kidney organoids [49]. Non-adhered renal organoids differentiation was carried out as follows. SOX9^+^ KPCs were seeded at 1 × 10^5^ cells/well in 96-well U-bottom low cell-binding plates (S-bio, Japan) for organoids formation. The medium was changed to organoids differentiation medium (Advanced DMEM/F12 medium supplemented with 1× l-Glutamin, 0.12 mM HEPES buffer, B27 additive (1:50, Gibco), N2 additive (1: 100. Gibco), 5 ng/mL insulin, 200 ng/mL human EGF, 100 ng/ml human FGF-10, 10 μL/mL human HGF, 1 mM N-acetyl-l-cysteine, 40 mM nicotinamide, 2 μg/mL hydrocortisone, 10 μM Y-27632, and 1% penicilin/streptomycin). After 7 days of culture, the organoids were harvested and performed immunofluorescence staining. Images were taken under a fluorescence microscope (Olympus IX73, Olympus, Japan).

### Immunofluorescence

For cell immunofluorescence staining, cells were fixed with 4% paraformaldehyde and then incubated with 0.25% Triton X-100 for 8 min to permeabilize. For tissue histology and immunofluorescence staining, tissue samples were fixed in 3.7% formaldehyde overnight at 4°C. For cryosection, the fixed tissue was infiltrated with 30% sucrose before embedding, then embedded into the Tissue-Tek O.C.T compound (Sakura, Japan), 5−8 μm sections were prepared using a cryotome (Leica Microsystem, Germany). For the paraffin section, the fixed tissue was dehydrated by ethanol gradient, processed in an automatic tissue processor, and then embedded into the paraffin blocks. All the samples were sectioned at 5−10 μm thickness using a microtome (Leica Microsystem). Haematoxylin and eosin (H&E) staining were performed following standard protocol. Immunofluorescence staining was conducted to reveal the protein expression in a standard protocol using the antibodies on paraffin sections, cryosections, or glass smears. Antibodies used for immunofluorescence include: SOX9 (1:200, CY5400, Abways), Ki67 (1:100, 550609, BD bioscience), ATP1A1 (1:50, sc-514614, Santa cruz), AQP1 (1:50, sc-25287, Santa cruz), SLC22A6 (1:200, ab183086, Abcam), Umod (1:200, ab207170, Abcam), AQP2 (1:200, ab199975, Abcam), Calbindin (1:200, 14479-1-AP, Proteintech), SLC12A3 (1:200, ab95302, Abcam), PAX8 (1:200, ab189249, Abcam), SYNPO (1:200, 21064-1-AP, Proteintech), GFP (1:200, ab290, Abcam), GFP (1:500, ab6673, Abcam), HuNu (1:200, ab191181, Abcam), and human specific Lamin A+C (1:200, ab108595, Abcam). Alexa Fluor-conjugated Donkey 488/594 (1:200, Life Technologies, USA) were used as secondary antibodies. In control experiments, the primary antibodies were replaced by IgG.

### Quantitative real time polymerase chain reaction

Total RNA was prepared from cultured uKPCs and organoids using a Rneasy Mini kit (QIAGEN) according to the manufacturer’s instructions. All RNA was digested with DNase I (Takara, Japan). 1 ug total RNA and PrimeScript RT Master Mix (Takara) was used for reverse transcription in a SimpliAmp Thermal Cycler (Life Technologies, USA). qRT-PCR was performed in triplicate using a QuantStudio3 Sequence Detection System and SYBR Premix Ex Taq II (Takara). DNA primer pairs were designed to span exons, when possible, to ensure that the product was from mRNA. The following cycling conditions were used: 1 cycle of 95°C for 30 s, 40 cycles of 95°C for 5 s, and 60°C for 34 s. The specificity of the amplified product was evaluated using the melting curve analysis. Internal control glyceraldehyde 3-phosphate dehydrogenase (GAPDH) was used to normalize the result in each reaction, and relative fold change was calculated by the 2−ΔΔCt method.

The following primer pairs were used:

**Table:** 

Species	Gene	Forward primer	Reverse primer
Human	*GAPDH*	AGTATGACAACAGCCTCAAGAT	GTCCTTCCACGATACCAAA
	*ATP1A1*	AGCTGCTCTGTGCTTTTCTCTC	ATACTTATCACGTCCAACCCCCTT
	*CDH1*	GACCGGTGCAATCTTCAAA	TTGACGCCGAGAGCTACAC
	*AQP4*	CAAGGCGGTGGGGTAAGTG	CATGGCCAGAAATTCCGCTG
	*SOX9*	GGCAAGCTCTGGAGACTTCTG	CCCGTTCTTCACCGACTTCC
	*KI67*	AAATTTGCTTCTGGCCTTCCC	TGTCACATTCAATACCCCTTCCA
Mouse	*GAPDH*	CGGAGTCAACGGATTTGGTCGTAT	AGCCTTCTCCATGGTGGTGAAGAC
	*SOX9*	AGCACAAGAAAGACCACCCC	ATGTGAGTCTGTTCCGTGGC
	*PAX2*	GGGAAGCTACCCTACCTCCA	TGCTGAATCTCCAAGCCTCA

### Microarray analysis, RNA-sequencing analysis, and bioinformatics

Mouse tissue, progenitor cells, and organoids were characterized by transcriptome microarrays (Affymetrix, USA) to study their gene expression profiles. Duplicate experiments for microarray were taken from two biological samples. Briefly, total RNA was checked for a RIN number to inspect RNA integrity by an Agilent Bioanalyzer 2100 (Agilent Technologies, Santa Clara, CA, USA). Double-stranded cDNA from samples were synthesized, labelled, and hybridized to Mouse Genome 430 2.0 array (Affymetrix, Santa Clara, CA, USA). The chips were scanned by GeneChip® Scanner 3000 (Cat#00-00212, Affymetrix) and Command Console Software 4.0 (Affymetrix) with default settings. Raw data were normalized by MAS 5.0 algorithm in R.

Human kidney tissue, kidney progenitors, and organoids were analysed by RNA sequencing. Duplicate experiments were taken from two biological samples. RNA was extracted using RNeasy® Mini Kit (REF# 74104), and 3 μg RNA per sample was used as input material for the RNA sample preparations. Sequencing libraries were generated using NEBNext® UltraTM RNA Library Prep Kit for Illumina® (NEB, USA) following the manufacturer’s recommendations, and index codes were added to attribute sequences to each sample. Raw data (raw reads) of FASTQ format were processed through in-house Perl scripts. In this step, clean data (clean reads) were obtained by removing reads containing adapter, reads containing ploy-N, and low-quality reads from raw data. At the same time, the clean data were calculated for Q20, Q30, and GC content. All the downstream analyses were based on clean data with high quality. FASTQ files were aligned against the human reference (hg19/hGRC38) genome. The index of the reference genome was built using STAR (v2.5.1b), and paired-end clean reads were aligned to the reference genome. HTSeq v0.6.0 was used to count the read numbers mapped to each gene, and then the FPKM of each gene was calculated based on the length of the gene and read counts mapped to this gene. Differential expression analysis of two conditions/groups (two biological replicates per condition) was performed using the DESeq2 R package (1.10.1). The resulting *P* values were adjusted using Benjamini and Hochberg’s approach for controlling the false discovery rate. Genes with an adjusted *P* value <0.05 found by DESeq2 were assigned as differentially expressed. Before differential gene expression analysis, the read counts were adjusted by edgeR (3.12.1) program package for each sequenced library through one scaling normalized factor. Differential expression analysis of two conditions was performed using the edgeR package. The *P* values were adjusted using the Benjamini & Hochberg method. A corrected *P* value of 0.05 and an absolute fold change of 2 were set as the threshold for significant differential expression. Heatmaps and principal component analysis (PCA) plots were generated in R. GO enrichment analysis of differentially expressed genes was implemented by the cluster Profiler R package, in which gene length bias was corrected. GO terms with corrected *P* value less than 0.05 were significantly enriched by differentially expressed genes.

### Single-cell RNA sequencing and bioinformatic analysis

#### Single cell sequencing

Single cells were captured and barcoded in 10× Chromium Controller (10× Genomics). Subsequently, RNA from the barcoded cells was reverse-transcribed, and sequencing libraries were prepared using Chromium Single Cell 3’ v3 Reagent Kit (10× Genomics) according to the manufacturer’s instructions. Sequencing libraries were loaded on an Illumina NovaSeq with 2 × 150 paired-end kits at Novogene, China. Raw sequencing reads were processed using the Cell Ranger v.3.1.0 pipeline from 10× Genomics. In brief, reads were demultiplexed and aligned to the human GRCh38 genome, and unique molecular identifiers counts were quantified per gene per cell to generate a gene-barcode matrix. Data were aggregated and normalized to the same sequencing depth, resulting in a combined gene-barcode matrix of all samples.

#### Quality control

Analysis of single-cell data was performed by R (version 4.0.5). R package Seurat (version 4.0.2) was used for quality control. We removed the low-quality cells with less than 200 or more than 9000 detected genes or if their mitochondrial gene content was >25%. Genes were filtered out that were detected in less than three cells. This filtering step resulted in 20205 genes × 5668 cells. We used monocle3 (version 1.0.0) for the subsequent analysis.

#### Dimensionality reduction and cluster analysis

The previous Seurat project generated the CDS file. We used the ‘preprocess_cds’ and ‘reduce_dimension’ methods to normalize the data and performed a dimensionality reduction of PCA. We performed PCA dimensionality reduction with the default number of genes and selected the 100 principal components for the ‘preprocess_cds’ method. We used the ‘cluster_cells’ method in monocle3 to identify cell clusters. The function FindAllMarkers performed differential expression analysis in Seurat to identify the marker genes with the likelihood-ratio test. Differentially expressed genes expressed at least in 25% of cells within the cluster and with a fold change of more than 0.25 (log scale) were considered marker genes. The function Featureplot performed featureplots in Seurat with the default parameters. The threshold of legends was adjusted with the ggplot2 (version 3.3.6) R package. StackedVlnPlot was implemented in Python by using scanyuan and scanpy methods.

#### Integration analysis

Single-cell co-analysis with iPSC-derived kidney organoids was achieved through the Seurat package in the R. After importing the KPC-derived organoids data and iPSC-derived kidney organoids data separately, quality control was performed, and low-quality cells were screened out individually. *FindIntegrationAnchors()* and *IntegrateData()* functions were used for removing batch effects and integrating two types of data. The subsequent analysis process is consistent with scRNA-seq analysis.

#### Trajectory analysis

Single-cell pseudotime trajectory was constructed by Monocle3 (version 1.0.0) R package. Cells from clusters ‘PT-like’, ‘LHDT-like’, ‘CD-like’, ‘Epithelial cell’, and ‘Progenitor cell’ were selected for analysis. The learn graph function was used to learn the trajectory graph. The cells were ordered along the trajectory using the *order_cells()* function. The *plot_genes_in_pseudotime*() function was used to show that the gene expression varies with the pseudotime series.

#### GO analysis

GO enrichment analysis of differentially expressed genes was implemented by the ClusterProfiler (version 3.18.1) R package. GO terms with a corrected *P* value less than 0.05 were considered to be significantly enriched. Bar plots were used to visualize enriched terms by the enrichplot (version 1.10.2) R package.

#### GSEA analysis

We used the clusterProfiler package for the pathway enrichment analysis of GSEA. The gene expression matrix that was differentially expressed between the two groups was sorted according to foldchange, representing the gene expression trend between the two groups. GSEA analyzes whether all the genes in a gene set are enriched at the top or bottom of the ranked list, and if they are enriched at the top, the gene set is up-regulated, and conversely, if they are enriched at the bottom, then is a down-regulation trend.

### Research ethics

All institutional and national guidelines for the care and use of laboratory animals were followed. All procedures followed were in accordance with the ethical standards of the responsible committee on human experimentation (institutional and national) and with the Helsinki Declaration of 1975, as revised in 2000. Informed consent was obtained from all individuals for being included in the study.

### Statistical analysis

Block randomization was used to randomize mice and samples into groups of similar sample sizes. No samples or animals were excluded from all analyses. Statistical power analysis was used to ensure adequate sample size for detecting significant differences between samples. At least two blinded participating investigators assessed all experiments. Results are presented as means ± standard error of the mean (SEM). GraphPad Prism (version 7.0a), FlowJo 10.0.8r1, Adobe Photoshop CS6, Adobe Premiere Pro CS6 v6.0, ImageJ (version 1.53t), and R programming were used for data management, statistical analysis, and graph generation. Paired results were assessed using parametric tests such as the Student’s *t*-test. Comparisons between multiple groups were analysed using one-way, or two-way ANOVA followed by Bonferroni’s post hoc test, and differences with *P* ≤ 0.05 were considered statistically significant.

### Data availability

The data that support the findings of this study are available from the corresponding author upon reasonable request. scRNA-seq data and codes for renal tubular organoids were uploaded to Github. github.com/Cheems97/Renal-tubular-organoids.git

## Supplementary Material

lnad047_suppl_Supplementary_Figure_S1

lnad047_suppl_Supplementary_Figure_S2

lnad047_suppl_Supplementary_Figure_S3

lnad047_suppl_Supplementary_Figure_S4

lnad047_suppl_Supplementary_Table_S1

lnad047_suppl_Supplementary_Data
