# Supplementary figures and images for: Generation of renal tubular organoids from adult SOX9+ kidney progenitor cells
Source: Life Med. 2023 Nov 23;2(6):lnad047. doi: 10.1093/lifemedi/lnad047 (PMC11749593; doi:10.1093/lifemedi/lnad047)

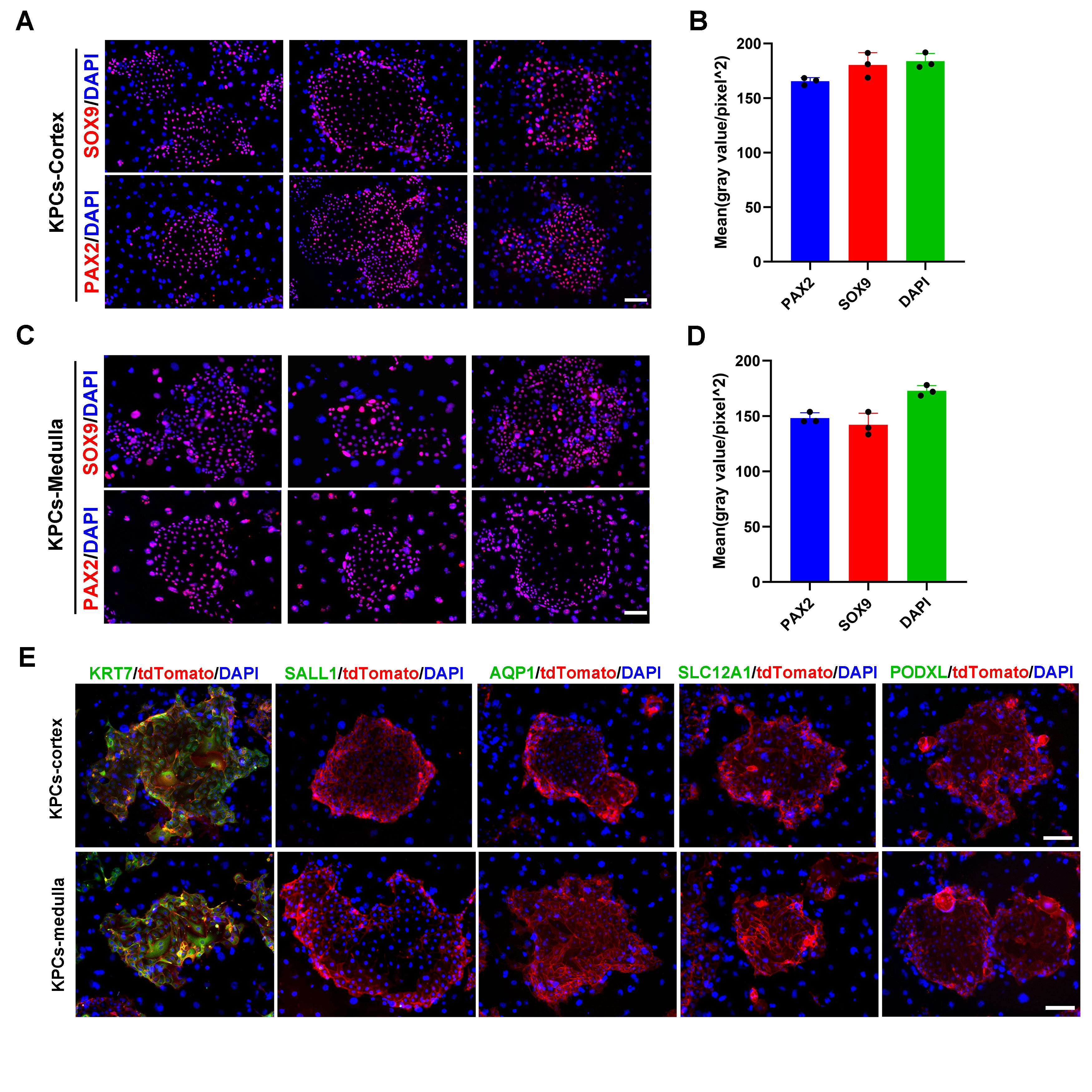

Supplement: lnad047_suppl_Supplementary_Figure_S1 [file lnad047_suppl_Supplementary_Figure_S1.tif]

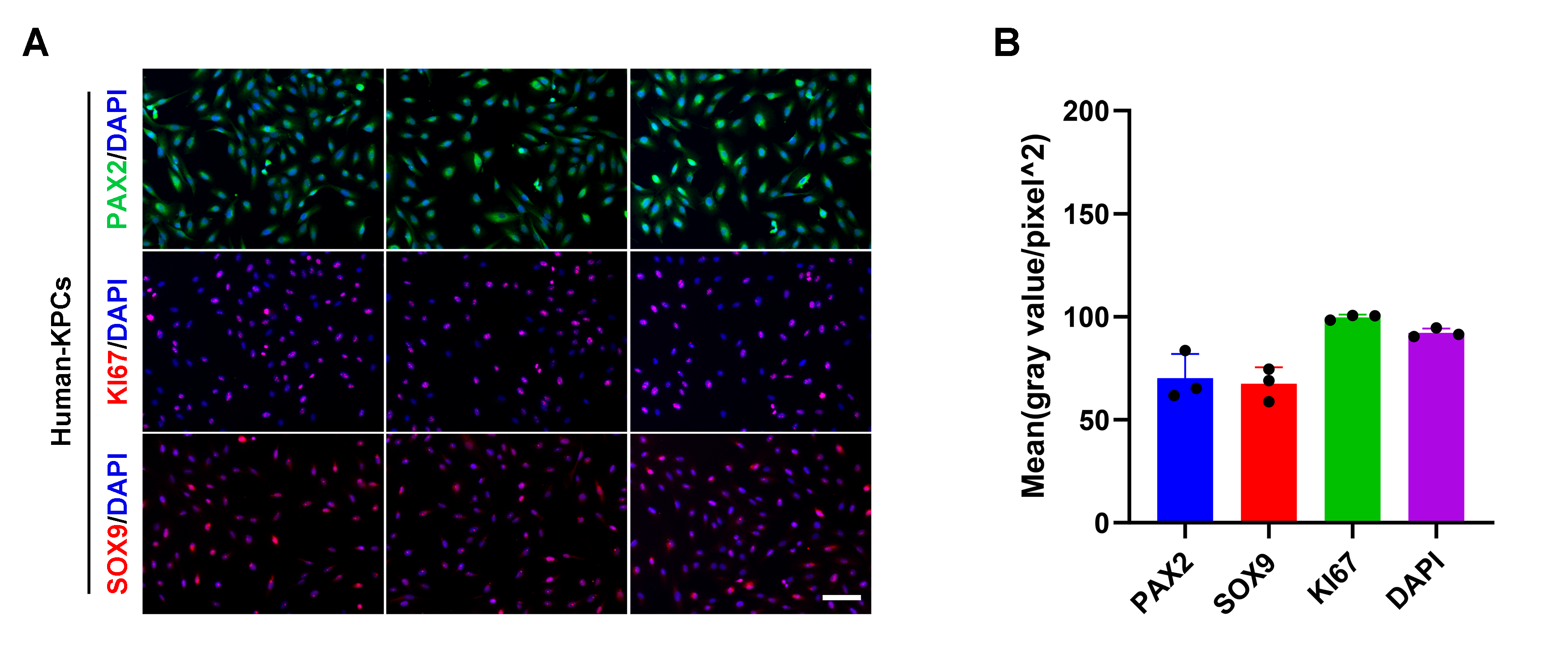

Supplement: lnad047_suppl_Supplementary_Figure_S2 [file lnad047_suppl_Supplementary_Figure_S2.tif]

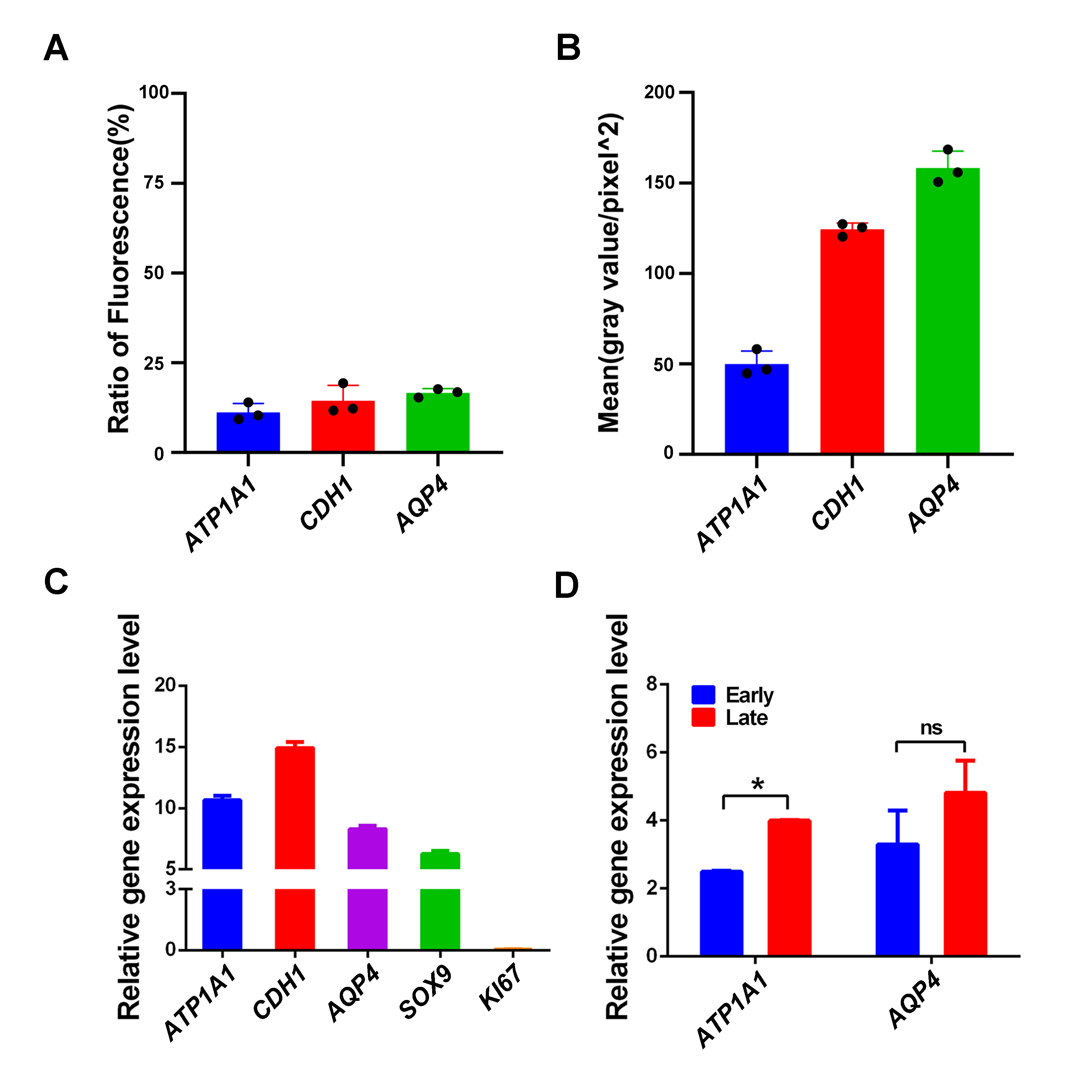

Supplement: lnad047_suppl_Supplementary_Figure_S3 [file lnad047_suppl_Supplementary_Figure_S3.tif]

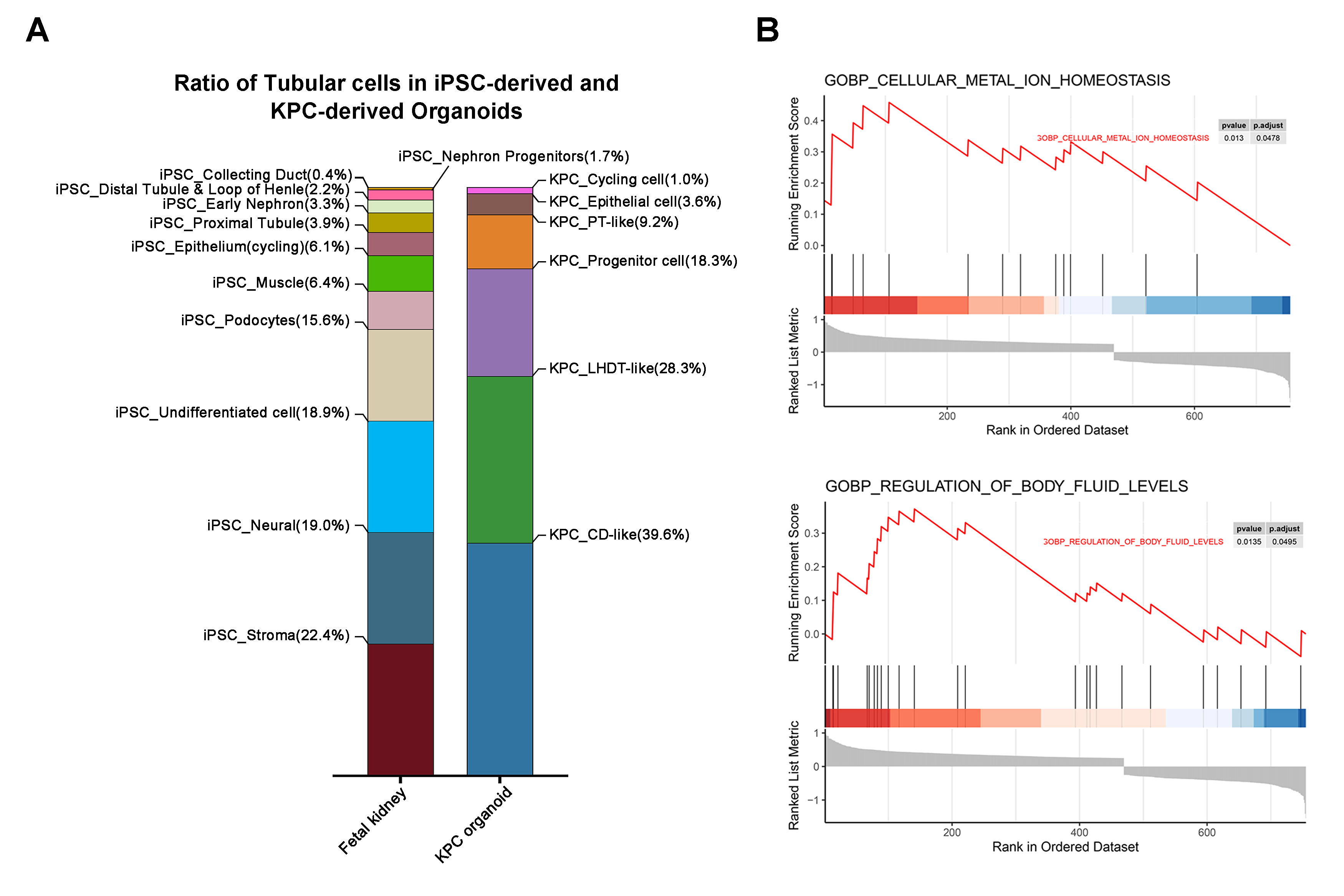

Supplement: lnad047_suppl_Supplementary_Figure_S4 [file lnad047_suppl_Supplementary_Figure_S4.tif]
