# Supplementary material for: Generation of renal tubular organoids from adult SOX9+ kidney progenitor cells
Source: Life Med. 2023 Nov 23;2(6):lnad047. doi: 10.1093/lifemedi/lnad047 (PMC11749593; doi:10.1093/lifemedi/lnad047)
Supplement: lnad047_suppl_Supplementary_Table_S1 [file lnad047_suppl_Supplementary_Table_S1.pdf]

Supplementary Table 1 | The characteristic genes used to define cluster types.

**Serial number**	**Gene names**	**The types of tissue defined in the cited articles**	**The types of clusters defined in this paper**
1	*VCAM1*	Proximal tubular cell	PT-like cell
2	*CDH6*	Proximal tubular cell	PT-like cell
3	*SLC12A1*	Loop of Henle cell, Distal tubular cell	LHDT-like cell
4	*PPP1R1B*	Loop of Henle cell	LHDT-like cell
5	*IGFBP5*	Collecting duct cell	CD-like cell
6	*PAX8*	Epithelial cell	Epithelial cell
7	*CENPF*	Cycling cell	Cycling cell
