# Supplementary material for: Generation of renal tubular organoids from adult SOX9+ kidney progenitor cells
Source: Life Med. 2023 Nov 23;2(6):lnad047. doi: 10.1093/lifemedi/lnad047 (PMC11749593; doi:10.1093/lifemedi/lnad047)
Supplement: lnad047_suppl_Supplementary_Data [file lnad047_suppl_Supplementary_Data.docx]

**Supplementary Figures**

**Figure S1.** **Immunofluorescence staining of mouse KPCs.**

(A) Images of mouse cortex-derived KPCs immunofluorescence staining for SOX9 and PAX2. (Scale bar: 50 μm) (B) Mean fluorescence intensity analysis of mouse cortex-derived KPCs immunofluorescence staining for SOX9 and PAX2. (*n* = 3 independent immunofluorescence experiments). Data are presented as means ± standard deviation (SD). (C) Images of mouse medulla-derived KPCs immunofluorescence staining for SOX9 and PAX2. (Scale bar: 50 μm). (D) Mean fluorescence intensity analysis of mouse medulla-derived KPCs immunofluorescence staining for SOX9 and PAX2. (*n* = 3 independent immunofluorescence experiments). Data are presented as means ± SD. (E) Images of mouse KPCs immunofluorescence staining for KRT7, SALL1, AQP1, SLC12A1, PODXL. Cells were derived from tdTomato-expressing mice. (Scale bar: 50 μm)

**Figure S2**. **Immunofluorescence staining of human KPCs.**

(A) Images of human KPCs immunofluorescence staining for SOX9, PAX2 and KI67. (Scale bar: 50 μm) (B) Mean fluorescence intensity analysis of human KPCs immunofluorescence staining for SOX9, PAX2 and KI67. (*n* = 3 independent immunofluorescence experiments). Data are presented as means ± SD.

**Figure S3**. **Statistical analysis of immunofluorescence and PCR data of human organoids.**

(A) Mean fluorescence area analysis of human organoids immunofluorescence staining for ATP1A1, CDH1 and AQP4. (*n* = 3 independent immunofluorescence experiments). Data are presented as means ± SD. (B) Mean fluorescence intensity analysis of human organoids immunofluorescence staining for ATP1A1, CDH1 and AQP4. (*n* = 3 independent immunofluorescence experiments). Data are presented as means ± SD. (C) Quantitative mRNA analysis of the above markers in organoids. (D) RT-qPCR analysis depicted related expressions of mature tubular markers in the early and late passage (*n* = 3 independent RT-qPCR experiments). Statistics are inclusive of all biological replicates. Data are presented as means ± SD.* *P* < 0.05.

**Figure S4**. **Characterization of tubular-like clusters in KPC-derived organoids.**

(A) Bar graph of cell count presenting the contribution of different clusters of nephrogenic cells to iPSC-derived organoids and KPC-derived organoids. (B) Gene Set Enrichment Analysis of human organoids tubule-like clusters. The red curve represents the upward adjustment of the pathway.
